# Different Head-Sway Responses to Optic Flow in Sitting and Standing With a Head-Mounted Display

**DOI:** 10.3389/fpsyg.2020.577305

**Published:** 2020-10-06

**Authors:** Kanon Fujimoto, Hiroshi Ashida

**Affiliations:** ^1^Department of Psychology, Graduate School of Letters, Kyoto University, Kyoto, Japan; ^2^Japan Society for the Promotion of Science, Tokyo, Japan

**Keywords:** virtual reality, optic flow, self-motion, vection, sitting, postural control, HMD

## Abstract

We investigated postural responses (head displacements) and self-motion perception (vection) to radial and lateral optic flows while sitting and standing by using a head-mounted display. We found that head displacement directions varied across postures. In the standing posture, radial optic flow generally produced the opposed head displacement against the perceived vection direction, consistent with the literature; however, in the sitting posture, the optic flow generally produced the following head displacement in the vection direction. In the standing posture, responses were evident soon after the onset of the optic flow presentation but became less clear in the latter half of a trial. The results, while less clear for lateral flows, were similar for both flow types. Our findings suggest partially distinct processes underlying vection and postural control.

## Introduction

Humans use multisensory sources of information, including vision, vestibular input, and proprioception, to perceive self-motion and control their bodies (Berthoz et al., 1975; Lee and Lishman, 1975; Wong and Frost, 1981; Bronstein, 1986; Day et al., 1997; Ash et al., 2011). Vision is especially crucial in postural control; when standing observers are exposed to a large area of coherent visual motion, known as optic flow, their bodies sway along the stimulus motion (Lishman and Lee, 1973; Lee and Aronson, 1974; Lee and Lishman, 1975; Lestienne et al., 1977; Dichgans and Brandt, 1978; Berthoz et al., 1979). This visually evoked postural response (VEPR) is considered to stabilize the body against self-motion (Lishman and Lee, 1973; Lestienne et al., 1977). Expanding and contracting optic flows simulate forward and backward self-motion, respectively, and backwards and forward postural sway would be induced to compensate. Likewise, rightward and leftward optic flows simulating leftward and rightward self-motion would be compensated by rightward and leftward postural sways, respectively. Vision also plays a dominant role in one’s conscious self-motion perception. The illusory self-motion perception induced by optic flow is referred to as vection (Fischer and Kornmüller, 1930).

Visual dominance in postural control has been considered in virtual reality (VR) technology, especially in recent cost-effective VR head-mounted displays (HMDs). Head motion measures through the HMDs, which represent postural responses, provide an effective tools to investigate user experience in VR scenes. Specifically, head movement could be an objective vection measure along with conventional subjective reports. For example, vection strength and VEPR magnitude change consistently when the intensity of visual stimuli is modified (Lestienne et al., 1977; Kuno et al., 1999; Kawakita et al., 2000; Lubeck et al., 2015). Also, VEPR magnitude is greater in periods of vection than in periods without vection (Thurrell and Bronstein, 2002; Freitas Júnior and Barela, 2004; Tanahashi et al., 2007; Guerraz and Bronstein, 2008). Considering possible confounds with cognitive biases (see discussion in Palmisano et al., 2015), subjective measures should be better complemented by more objective measures.

Although VR devices could be used while sitting in daily situations, it remains unclear how VEPR is effectively induced in stably sitting observers. Given the changes in the body’s balance, proprioceptive feedback, and linear vection modulation (Guterman et al., 2012), it is crucial to understand postural and vection responses under both sitting and standing postures, as visual and vestibular inputs are similar whereas proprioceptive inputs may differ (Genthon and Rougier, 2006). In the standing posture, afferent inputs from the ankle joints and leg muscles are critical in detecting postural sway (Fitzpatrick and McCloskey, 1994) and maintaining an upright posture (Lee and Lishman, 1975; Dietz et al., 1992). In the sitting posture, buttock and thigh cutaneous receptors and hip receptors can replace ankle joint inputs to control posture (Genthon and Rougier, 2006). Despite such differences, vision can still affect sitting postural control. For example, Mizuno et al. (2001) reported that the body sways with visual oscillation when sitting on an unstable board. Since their participants were seated on an unstable board, VEPR in more natural stable sitting remains unexplored – a literature gap that this study intends to fill.

Thus, in this study, we investigated VEPR and vection in sitting and standing postures. For visual stimulation, we used an HMD to present radial (expansion and contraction) and lateral (rightward and leftward) optic flows of random dots. We measured the head displacement obtained from the HMD to compare postural responses while sitting and standing. Postural sway is often measured by the center of foot pressure (CoP) and head displacement. However, for the purpose of this study we believe that head displacement alone would be a satisfactory measure of VEPR magnitude. In the standing posture, CoP and head mostly move in the same direction while observing visual motion (Tanahashi et al., 2007; Guerraz and Bronstein, 2008), and it is unlikely that they move more independently in the sitting posture given the physical constraints.

## Method

### Participants

The participants consisted of 19 adults (11 females, 8 males; mean age = 21.90 years; standard deviation [SD] = 1.55) from Kyoto University with normal or corrected-to-normal visual acuity and with no history of vestibular disorders. They were not informed about the purpose of the study. All of them provided written informed consent to undergo the experimental procedure, following the ethical standards of the Declaration of Helsinki, and the study was approved by Kyoto University’s Ethics Committee.

### Apparatus and Stimuli

Stimuli were presented through an Oculus Rift CV1 HMD (Oculus VR, Irvine, CA, United States; refresh rate = 90 Hz, resolution = 1080 × 1200 pixels/eye), with an estimated 110° field of view (FoV) for each eye, measured diagonally. The lateral distance of the Rift’s lenses was adjusted according to each participant’s interpupillary distance. HMD position and orientation were recorded at 50 Hz using the HMD’s tracking system. The experiment was controlled by a PC running Windows 10 (Microsoft, Redmond, WA, United States) with a Core i7-6700 CPU (Intel, Santa Clara, CA, United States) and a GTX 1060 graphics card (NVIDIA, Santa Clara, CA, United States). An Xbox One gamepad (Microsoft) was used to collect participant responses.

**FIGURE 1:**
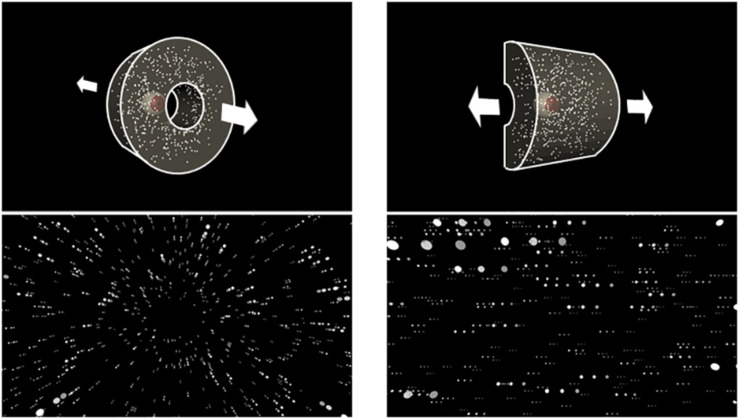
Schematic structures of the three-dimensional cloud (upper panels) and optic flow stimulus generated by the camera movement in each structure (lower panels). The upper left panel shows the eye representing the virtual camera moving through the central hole, generating radial optic flow (lower left panel). In the upper right panel, the eye moved laterally along the half-cylinder, producing lateral optic flow (lower right panel). Notably, the bottom panels show dots in several successive frames to illustrate motion.

A virtual environment was created using 3D Unity Engine 5.3.1 (Unity Technologies, San Francisco, CA, United States). In this environment, a 3-D cloud with 1,500 white spheres was presented against a black background. Each sphere’s simulated size in the virtual environment was 0.1 m, and the cloud structure depended on the direction of self-motion (Figure 1). Visual stimuli were viewed binocularly with simulated binocular disparities.

To simulate self-motion in depth, the spheres were presented as a virtual cylindrical cloud (radius = 25 m; length = 22 m). In order to avoid the participant making dodging postural responses (to potential collisions with objects), the spheres were not distributed within a 2 m radius from the cylinder’s central axis. The virtual camera’s initial viewpoint was at one end of the cylinder and was oriented toward the other end. The displays simulated either forward or backward self-motion along the axis of the cylinder at a constant velocity of ± 9.4 m/s. As a result, each eye was shown an expanding or contracting optic flow with binocular disparities. The spheres were always distributed within a distance of 0.01–22 m from the viewpoint. Once a sphere was out of this range, it randomly reappeared at the opposite edge of the range. At any frame, about 518 spheres were visible within the HMD’s FoV. The spheres’ retinal size continually changed from 0.5° to 2.4° as they approached or receded from the viewpoint. This radial optic flow condition was almost the same as that in Bubka et al. (2008), namely, in speed, dot size, and density.

To simulate leftward or rightward self-motion, the spheres were presented as a half-cylindrical cloud (radius = 25 m; length = 50 m). To prevent them from appearing too close to the eyes, no sphere was presented within 2 m from the cylinder’s axis. The initial viewpoint was in the middle of the cylinder and was oriented toward its curved surface. The displays simulated either a rightward or leftward camera movement along the cylinder’s axis at a constant velocity of ± 9.4 m/s. Therefore, each eye was presented either a leftward or rightward lateral optic flow with binocular disparities. Each sphere’s retinal size varied from 0.5° to 3.2°, and the spheres were always distributed within a range of 25 m right to 25 m left from the viewpoint. Once a sphere went outside this range, it reappeared randomly at the opposite edge of the range. Approximately 456 spheres were visible on the display monitor. The near (nearest boundary from which objects start to be presented) and far clipping planes (farthest boundary at which objects are no longer drawn) were 0.01 m and 22 m, respectively.

### Procedure

Because of the limited time of the experiment for the health and safety, we used two trials per condition for each participant. Two eight-trial blocks were conducted at two trials for each of the four optic flow directions. In one block, participants sat on a stable stool and held a gamepad on their lap with both hands. In the other block, they stood with their feet together, holding the gamepad in front of their body with both hands. They wore shoes in both blocks. The sequence of the conditions of optic flow and the blocks were randomized and counterbalanced across participants, respectively. Before proceeding with the main experiment, the participants performed several trials of an identical task in the first block until they were familiar with it. The whole experiment including the instruction and practice took no less than an hour for each participant.

To minimize the previous trial’s residual effects, a dark background (identical to the optic flow background) was presented for 30 s before each trial. Afterward, two white disks (with diameters of 3.3° and 1.8°) appeared in front of the displays. The large disk was fixed at the center of the virtual environment, and the small disk was fixed at the HMD’s center, which moved with the participant’s head. The participants were instructed to hover the small disk over the large one by moving their heads to orient their viewpoints straight ahead. If the participant successfully maintained the angular distance of the centers of the two pointers at less than 1° for 3 s, the two disks disappeared, and the next trial started automatically. After 1 s of presenting only the dark background, the optic flow stimulus was presented for 30 s. The participants’ main task during the optic flow observation was to continue pressing a button while they experienced vection. They were also instructed to keep their heads as upright as possible during the optic flow presentation to avoid any undesirable intentional movement. If either pitch or yaw orientation exceeded 3° upfront, the participants were warned by a beep through the built-in headphones. To mask room noise that could provide positional cues, a pink noise of approximately 62 dB SPL was presented through the headphones during the optic flow presentation.

After a 30 s presentation of optic flow, the participants reported the overall magnitude of vection on a scale of 0 to 100 using a directional pad. The rating criteria were adapted from Bubka et al. (2008). A value of 0 indicated that the participants felt stationary, and only the spheres appeared to move, while a value of 100 indicated that they felt as if they were moving through the stationary random spheres. After the magnitude rating, participants reported the vection direction using a five-point scale: forward, slightly forward, neither, slightly backward, and backward for radial optic flow and leftward, slightly leftward, neither, slightly rightward, rightward for lateral optic flow.

## Results

### Head Displacement

We separately examined the participants’ anterior–posterior (A/P) and medial–lateral (M/L) head movements. Because our focus was on the VEPR, we analyzed the A/P and M/L data for the expansion/contraction and rightward/leftward conditions, respectively.

**FIGURE 2:**
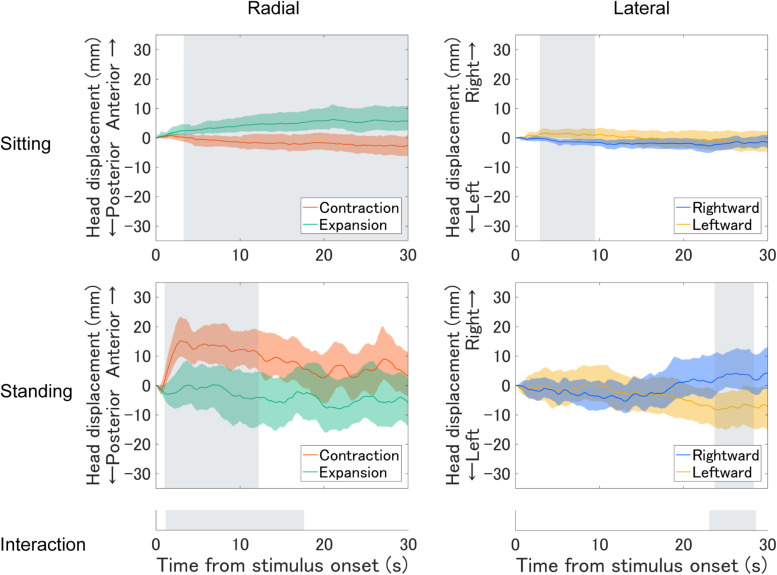
Mean time series of head displacement relative to the base position throughout the optic flow presentation for each posture. The top and middle panels plot the sitting and standing posture results, respectively. The left panels plot the findings for the expansion/contraction optic flow, and the positive and negative values along the vertical axis represent anterior and posterior displacements, respectively. The right panels plot the results for leftward/rightward optic flow, and the positive and negative values represent rightward and leftward displacements, respectively. Ribbons represent 95% confidence intervals obtained through the bootstrap method. Shaded regions in the top and middle panels highlight the period where a significant cluster was found with the cluster-based permutation test for expansion–contraction and rightward–leftward comparisons for each posture. The bottom panels highlight the period where a significant cluster was found for a two-way interaction between optic flow and posture in each radial and lateral condition.

First, we plotted the mean time series of head displacement relative to mean head position during the first 1 s before the optic flow presentation (base position; Figure 2). We computed confidence intervals (95%) using a bias-corrected and accelerated (BCa) bootstrap method (Efron, 1987). Under radial optic flow, the sitting participants tended to move their heads forward when they observed expansion and tended to slightly move backward when they observed contraction. In contrast, the standing participants tended to move their heads forward with contraction and tended to slightly move backward with expansion. In the standing posture, this tendency was evident soon after onset but became less clear in the latter half. Under lateral optic flow, the head displacement to the optic flow was less clear, but the patterns appeared similar. The sitting participants tended to slightly move their heads rightward or leftward when they observed leftward or rightward motion, respectively; the standing participants exhibited the opposite pattern. In the standing posture, the responses to the lateral optic flows were evident in a later period.

The head displacement in the orthogonal axis (i.e., M/L for radial flows and A/P for lateral flows) showed no directionally specific responses. Therefore, we will not discuss head displacement orthogonal to the simulated self-motion direction.

To statistically support the above findings, we tested the effects of optic flow and posture using a cluster-based permutation analysis (Maris and Oostenveld, 2007) separately for each optic flow condition (radial/lateral). Compared to summary statistical tests such as analysis of variance (ANOVA) on the average head position, this is a more direct approach to compare time courses across conditions, with the advantages of addressing multiple comparisons and avoiding arbitrary selection of time periods. The general procedure was as follows. First, we performed a two-tailed paired *t* test between conditions for each sampling point. Then we summed significant *t*-values (uncorrected *p* < 0.05) adjacent in time. If it formed several clusters of significant *t*-value sum, we selected the largest cluster. Then, we calculated the maximum cluster sum of the significant *t*-value for randomly permuted datasets 10,000 times. Consequently, we obtained a distribution of 10,000 maximum cluster sums produced by chance. Finally, we assessed if the cluster sum from the original datasets was significantly larger than those from the permuted datasets. The null hypothesis is that the data observed in the two conditions are drawn from the same probability distribution. If the original cluster sum was larger than 95% of the maximum clusters from the randomly permuted datasets (critical alpha = 0.05), we rejected the null hypothesis. We first evaluated the interaction across optic flow direction and posture. We then analyzed each effect more closely by comparing the two optic flow directions for each postural condition.

For the radial optic flow, we found a significant interaction across optic flow direction and posture (*p* < 0.01). Follow-up tests revealed significant differences between expansion and contraction for both sitting (*p* < 0.05) and standing (*p* < 0.05). For the lateral condition, we found a significant interaction between lateral optic flow and posture (*p* < 0.05). According to follow-up tests, significant differences existed between rightward and leftward directions for both sitting (*p* < 0.05) and standing postures (*p* < 0.05).

To examine the range of the head movement, we calculated the standard deviation (SD) of the sampled head positions in the relevant axes. In both radial and lateral conditions, the SD was larger in the standing than in the sitting posture, but there was no significant effect of optic flow or interaction (see Supplementary Material for details).

For further insights into the temporal dynamics of head displacement, we applied Detrended Fluctuation Analysis (DFA; Peng et al., 1995; Duarte and Zatsiorsky, 2001; Duarte and Sternad, 2008) to the head displacement data along the relevant axes. The scaling exponent α was between 1 and 1.5 under all conditions (see Supplementary Material), which indicates autocorrelation and non-stationarity in the signal (Peng et al., 1995; Hardstone et al., 2012). Under both radial and lateral conditions, α was smaller in the standing than in the sitting posture. This suggests more fractal structure retained in the standing posture, which might reflect larger instability and complexity of standing posture, as SD indicates.

In summary, posture determined the head displacements to optic flows. Particularly in the sitting posture, the head movement direction corresponded to the simulated self-motion, in contrast to the opposing head movement in the standing posture. In addition, standing posture produced more variability in head movement compared to the sitting posture (as expected).

### Vection Responses

**FIGURE 3:**
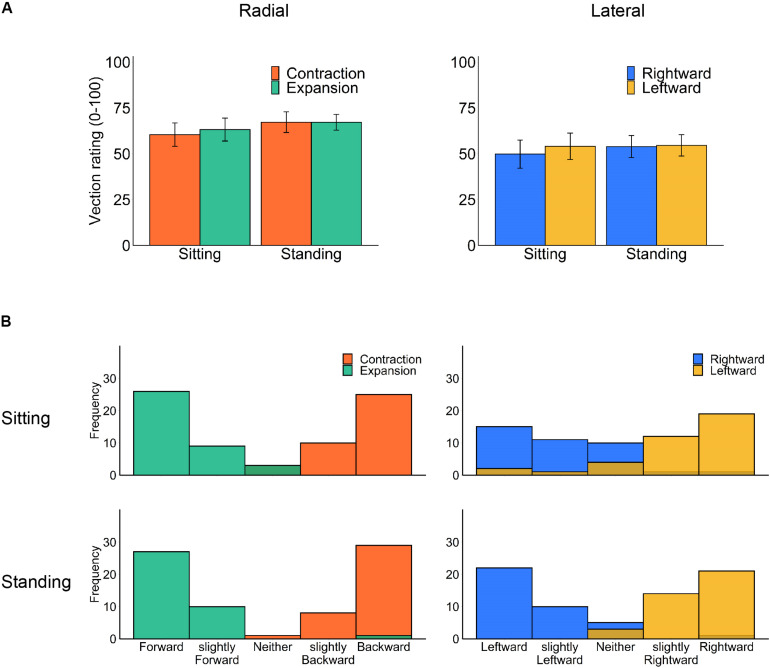
Vection results: **(A)** mean vection ratings and **(B)** histograms of perceived vection direction for each posture and optic flow type (left panels: radial optic flow [expansion/contraction], right panels: lateral optic flow [rightward/leftward]). Error bars show standard errors of the mean across participants.

We separately analyzed the rated strength of vection (Figure 3A) for each flow type under the two optic flow directions (contraction/expansion or leftward/rightward) and the two postural conditions (standing and sitting) using a two-way repeated-measures ANOVA. However, no significant main effect or interaction was observed (radial posture: *F*_1,18_ = 1.36, *p* = 0.259, η^2^ = 0.012; radial flow direction: *F*_1,18_ = 0.11, *p* = 0.740, η^2^ = 0.001; radial interaction: *F*_1,18_ = 0.10, *p* = 0.758, η^2^ = 0.001; lateral posture: *F*_1,18_ = 0.18, *p* = 0.675, η^2^ = 0.002; lateral flow direction: *F*_1,18_ = 0.31, *p* = 0.586, η^2^ = 0.002; lateral interaction *F*_2,18_ = 0.42, *p* = 0.524, η^2^ = 0.001).

We also computed the latency and duration from the online button responses (Table 1) but did not observe any significant main effect or interaction for either measure (all *p* > 0.05; see Supplementary Material).

**TABLE 1:** Mean vection latencies and durations for each posture and optic flow type.

	Expansion		Contraction		Rightward		Leftward	
	Sitting	Standing	Sitting	Standing	Sitting	Standing	Sitting	Standing
Latency (s)	7.58	5.98	7.01	6.16	11.14	9.16	10.94	11.03
Duration (s)	20.47	20.40	20.72	22.08	17.23	18.54	17.36	16.56

For comparing radial and lateral flows, we have collapsed the data of vection latencies, durations, and ratings for each flow type. All three measures consistently showed stronger vection for radial than lateral flow; paired *t* test revealed shorter latency (*t*_17_ = −3.33, *p* = 0.004, *d* = −0.461), longer duration (*t*_17_ = 2.97, *p* = 0.009, *d* = 0.407) and stronger vection rating (*t*_18_ = 2.84, *p* = 0.011, *d* = 0.539) for radial as compared with lateral flow.

Notably, our stimuli induced substantial vection under all conditions. One may suspect that contrasting head displacement is caused by a change in the perceived self-motion direction (Kuno et al., 1999). However, the reported vection direction after each trial was consistent with simulated self-motion in both sitting and standing postures (Figure 3B). Also, there was no significant correlation between vection responses (latency, duration, and rating) and the averaged head displacement for any condition (Supplementary Material), but this could be due to insufficient number of participants for correlation analysis.

## Discussion

### Summary

This study used a VR HMD to examine vection and VEPR (postural responses) while standing and sitting. While all conditions reliably induced vection, no remarkable difference in vection was observed across conditions. Most notably, the head displacements to optic flows were differently modulated across postures, as supported by the cluster-based permutation tests. The relative direction of head displacement to optic flow tended to reverse across sitting and standing. Head displacement while standing was consistent with the literature findings on VEPR, that is, displacement against perceived body motion (Lishman and Lee, 1973; Lee and Aronson, 1974; Lee and Lishman, 1975; Lestienne et al., 1977; Dichgans and Brandt, 1978; Berthoz et al., 1979). On the other hand, head displacement while sitting corresponded to the same vection direction – a novel finding in the current study.

Note that the patterns of fluctuation varied across conditions; the head displacement against vection direction was the most evident soon after the stimulus onset in the radial-standing condition, while the tendency was the most evident in the late period of a trial in the lateral-standing condition. Also, the processes are not stationary, as suggested by the DFA results. Although we focus on the dominant initial responses or the overall trends in the head displacement for the current purpose, VEPRs do not always occur in only one direction during the recording. Future studies should focus on the non-stationary temporal dynamics to reveal the complexity of VEPR.

### Head Displacement to Radial and Lateral Optic Flows

The results for the sitting posture cannot be explained using the conventional VEPR interpretation. VEPR while standing is thought to occur to compensate for an illusory fall (Lishman and Lee, 1973; Lestienne et al., 1977). For example, an expanding optic flow should provide a sense of leaning forward, leading to backward-sway compensation, and a contracting optic flow should induce a sense of leaning backward and produce a forward sway, corresponding to the current results for the standing posture. Swaying along with simulated self-motion does not compensate for but should rather facilitate falling. Therefore, sitting-posture responses might be operated independently of postural stabilization systems. Regarding this open question, further research may explore the mechanisms underlying different head displacements across postures.

We can speculate that sensory conflict might be related to the potential mechanisms of the sitting-postural control. Given that optic flow without physical motion produces visual-vestibular conflict that sometimes leads to visually induced motion sickness (Keshavarz et al., 2015), sitting participants might have moved toward the direction of simulated self-motion to produce vestibular signals and thus reduce visual–vestibular conflict consciously or unconsciously. We can assume that the postural stabilization and the sensory conflict mechanisms run in parallel, but the variable demand of maintaining posture might affect which mechanisms becomes dominant. As the SDs of head positions show, standing posture is less stable than the sitting posture. The imminent demand of maintaining unstable standing posture would allow more prominent responses for the postural stabilization. On the other hand, reduced demand for maintaining posture in the stable sitting posture could reduce the postural stabilization responses, thereby allowing more prominent responses for reducing sensory conflict.

Backward sways in the sitting–contraction and standing–expansion conditions were smaller than forward sways in the sitting–expansion and standing–contraction conditions. This small backward sway is consistent with the previous findings for a standing observer (Lestienne et al., 1977; Palmisano et al., 2009; Holten et al., 2013). Backward sway is thought to be smaller because the biomechanical constraint is more severe when pitching backward than forward, as our feet are attached forward, making it easier to lean forward than backward (Lestienne et al., 1977; Edwards and Ibbotson, 2007). The results for the sitting condition can be explained biomechanically as well: backward sway by contraction while sitting was smaller than forward sway by expansion because it is also easier for one to lean forward than backward when seated.

The buildup of the head displacement was different between radial-standing and lateral-standing conditions. Weaker vection responses for the lateral conditions compared to the radial conditions might be related to the later buildup of head displacement in the standing posture, as vection and postural sway are similarly modulated by visual parameters (Lestienne et al., 1977; Kuno et al., 1999; Kawakita et al., 2000).

Caution should be exercised in generalizing these findings because we only measured the head displacement. Although previous studies showed consistent movement of the head and CoP in the standing posture with visual motion (Tanahashi et al., 2007; Guerraz and Bronstein, 2008), it is not assured in the sitting posture, although they might move even more consistently in sitting given the reduced degree of freedom. Further investigation with full-body tracking would help understand the biomechanical dynamics of the postural control in the sitting posture.

### Vection Measurements

We did not observe significant differences in vection strength between the two postures, which is not consistent with Guterman et al. (2012), who reported higher vection strength in the sitting posture. This discrepancy, however, could be due to variations in experimental settings: the visual angle of the display in Guterman et al. (2012) was relatively small (39°). The participants could also have been made aware of their body sway by the motion of the visual aperture, which could attenuate vection, especially while standing. Our larger (110° diagonally) and more immersive display may have reduced body sway awareness, which in turn may have reduced vection strength differences between the two postures.

### Implications for Vr Applications

These results illustrate the usefulness of head displacement in a stable sitting posture in measuring VR user experience. Although many VR apps are used in the sitting posture, studies have not thoroughly investigated postural responses to visual motion while seated. Our findings on head displacement while sitting suggest that postural response could be an indicator of immersive experience (Palmisano et al., 2015). The current findings could also contribute to predicting visually induced motion sickness, as it is associated with greater likelihood of vection experience (Keshavarz et al., 2015). However, the relationships are still controversial (Palmisano et al., 2017; Kuiper et al., 2019). In addition, the implications of the opposite direction of head motion are open for further research. Non-linear analyses such as DFA and RQA, might be useful in predicting the sickness even in the sitting posture during the exposure to optic flow (Apthorp et al., 2014; Palmisano et al., 2018; Faust et al., 2019; Risi and Palmisano, 2019), emphasizing the importance of examining the temporal evolution of VEPRs in future research.

## Data Availability Statement

The datasets presented in this article are not readily available because of ethical restrictions on raw data availability. Requests to access the datasets should be directed to fujimoto.kanon.63a@st.kyoto-u.ac.jp.

## Ethics Statement

The studies involving human participants were reviewed and approved by The local ethics committee of Kyoto University. The patients/participants provided their written informed consent to participate in this study.

## Author Contributions

KF and HA reviewed and edited the manuscript. KF performed the experiments, analyzed the data, and wrote the manuscript with feedback from HA Meanwhile, HA and KF contributed to the study design. Both authors contributed to the article and approved the submitted version.

## Conflict of Interest

The authors declare that the research was conducted in the absence of any commercial or financial relationships that could be construed as a potential conflict of interest.
