# Supplementary material for: Different Head-Sway Responses to Optic Flow in Sitting and Standing With a Head-Mounted Display
Source: Front Psychol. 2020 Oct 6;11:577305. doi: 10.3389/fpsyg.2020.577305 (PMC7573131; doi:10.3389/fpsyg.2020.577305)
Supplement: Supplementary file 1 [file Data_Sheet_1.docx]

Supplementary Material

The results were analyzed by using a repeated-measures ANOVA, unless otherwise noted.

# Head displacement

Figure S1(a) shows the mean standard deviations (SD) of the head displacement across participants under the two conditions of optic flow and the two postural conditions. SD was first calculated for each trial, averaged for each participant, and averaged across participants. In the radial condition, the SD was calculated for the A/P axis, and in the lateral condition, the SD was calculated for the M/L axis.

For the radial conditions of optic flow, a significant main effect of posture (*F*_1,18_ = 60.74, *p* < 0.001, η^2^ = 0.511) was observed, but no significant main effect of optic flow (*F*_1,18_ = 0.003, *p* = 0.957, η^2^ < 0.001) or two-way interaction (*F*_1,18_ = 0.034, *p* = 0.856, η^2^ < 0.001) was found. For the lateral conditions of optic flow, a significant main effect of posture (*F*_1,18_ = 44.40, *p* < 0.001, η^2^ = 0.411) was observed, but no significant main effect of optic flow (*F*_1,18_ = 0.02, *p* = 0.903, η^2^ < 0.001) or two-way interaction (*F*_1,18_ = 1.15, *p* = 0.298, η^2^ = 0.005) was found.

Figure S1(b) shows the mean exponent *α* from the Detrended Fluctuation Analysis of the head displacement across participants for each optic flow and posture, by using a MATLAB script of Wenye (2020). In the radial condition, *α*s were calculated for the A/P axis, and in the lateral condition, *α*s were calculated for the M/L axis. For the radial conditions of optic flow, a significant main effect of posture (*F*_1,18_ = 4.92, *p* = 0.040, η^2^ = 0.064) was observed, but no significant main effect of optic flow (*F*_1,18_ = 0.50, *p* = 0.491, η^2^ = 0.004) or two-way interaction (*F*_1,18_ = 0.14, *p* = 0.713, η^2^ = 0.001) was found. For the lateral conditions of optic flow, a significant main effect of posture (*F*_1,18_ = 5.26, *p* = 0.034, η^2^ = 0.078) was observed, but no significant main effect of optic flow (*F*_1,18_ = 3.76, *p* = 0.068, η^2^ = 0.039) or two-way interaction (*F*_1,18_ = 1.64, *p* = 0.216, η^2^ = 0.011) was found.


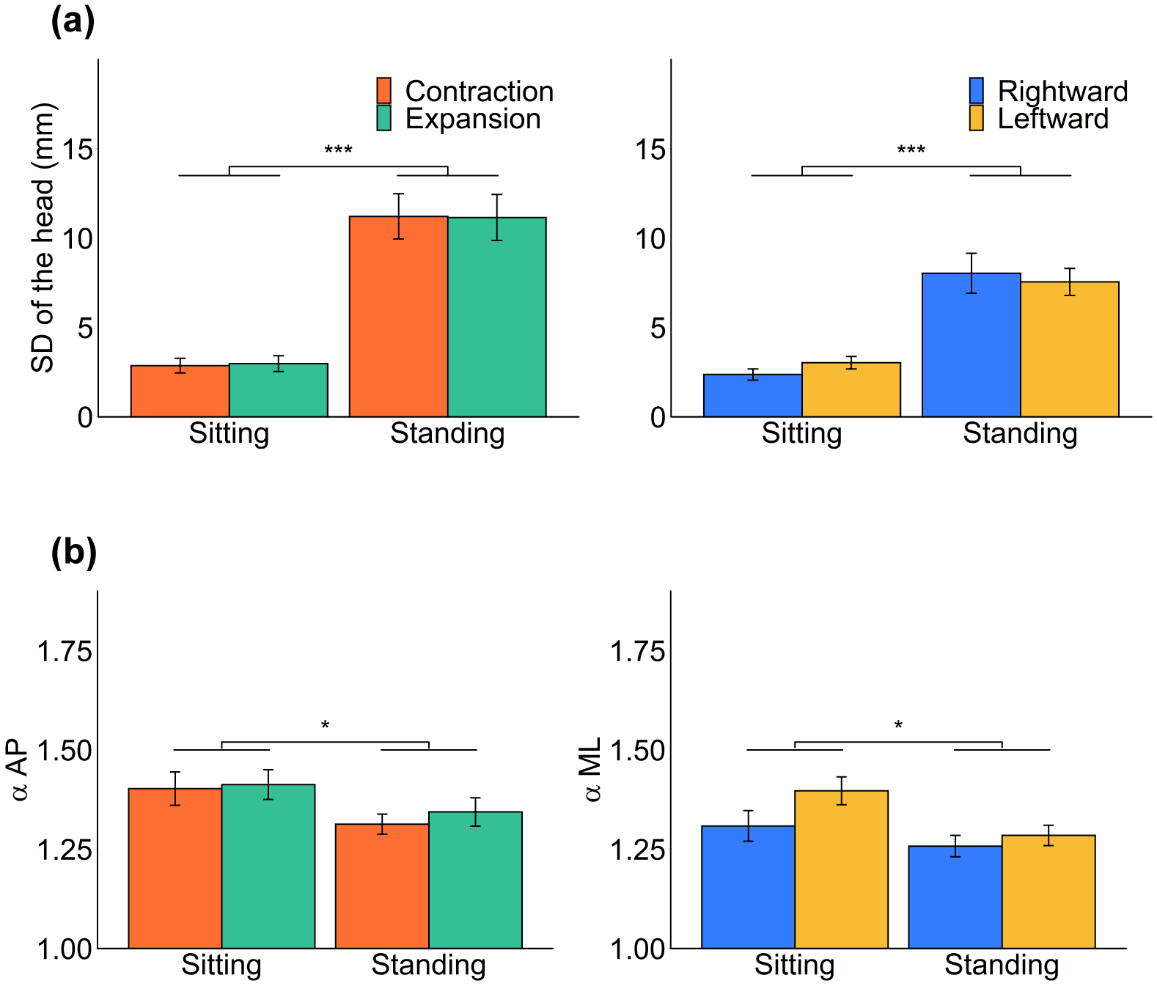


**Figure S1.** (a) Standard deviation of the head position and (b) *α* exponent from the Detrended Fluctuation Analysis for each posture and condition of optic flow (left panel: expansion/contraction, right panel: rightward/leftward), with standard errors of mean. * and *** indicates the statistical significance < .05 and < 0.001, respectively.

# Vection measures

The online response data for one participant were excluded from the analyses because an incorrect button was used for the online reports.

Figure S2(a) shows the mean vection latencies across participants under the two conditions of optic flow and the two postural conditions. For the radial conditions of optic flow, no significant main effect (posture: *F*_1,17_ = 1.62, *p* = 0.220, *η*^2^ = 0.010; optic flow: *F*_1,17_ = 0.02, *p* = 0.901, *η*^2^ = 0.000) or two-way interaction (posture and optic flow: *F*_1,17_ = 0.11, *p* = 0.747, *η*^2^ = 0.001) was observed. For the lateral conditions of optic flow also, no significant main effect (posture: *F*_1,17_ = 0.35, *p* = 0.560, *η*^2^ = 0.003; optic flow: *F*_1,17_ = 0.54, *p* = 0.474, *η*^2^ = 0.002) or two-way interaction (*F*_1,17_ = 0.86, *p* = 0.367, *η*^2^ = 0.003) was observed.

Figure S2(b) shows the mean vection durations across participants under the two conditions of optic flow and two postural conditions. For the radial conditions of optic flow, no significant main effect (posture: *F*_1,17_ = 0.26, *p* = 0.614, *η*^2^ = 0.002; optic flow: *F*_1,17_ = 0.48, *p* = 0.496, *η*^2^ = 0.004) or two-way interaction (*F*_1,17_ = 0.29, *p* = 0.597, *η*^2^ = 0.002) was observed. For the lateral conditions of optic flow also, no significant main effect (posture: *F*_1,17_ = 0.03, *p* = 0.862, *η*^2^ = 0.000; optic flow: *F*_1,17_ = 0.42, *p* = 0.523, *η*^2^ = 0.002) or interaction (*F*_1,17_ = 1.14, p = 0.301, *η*^2^ = 0.003) was observed.

Table S1 shows the correlation matrix of vection responses and the mean head displacement. Mean head displacements were calculated for each trial as the mean head displacement during the presentation of optic flow relative to the initial position of the head. We then averaged the mean head displacement for each participant and condition. Family-wise errors were controlled with the Holm–Bonferroni method. We found no significant correlation for any combinations of vection and the head displacement.


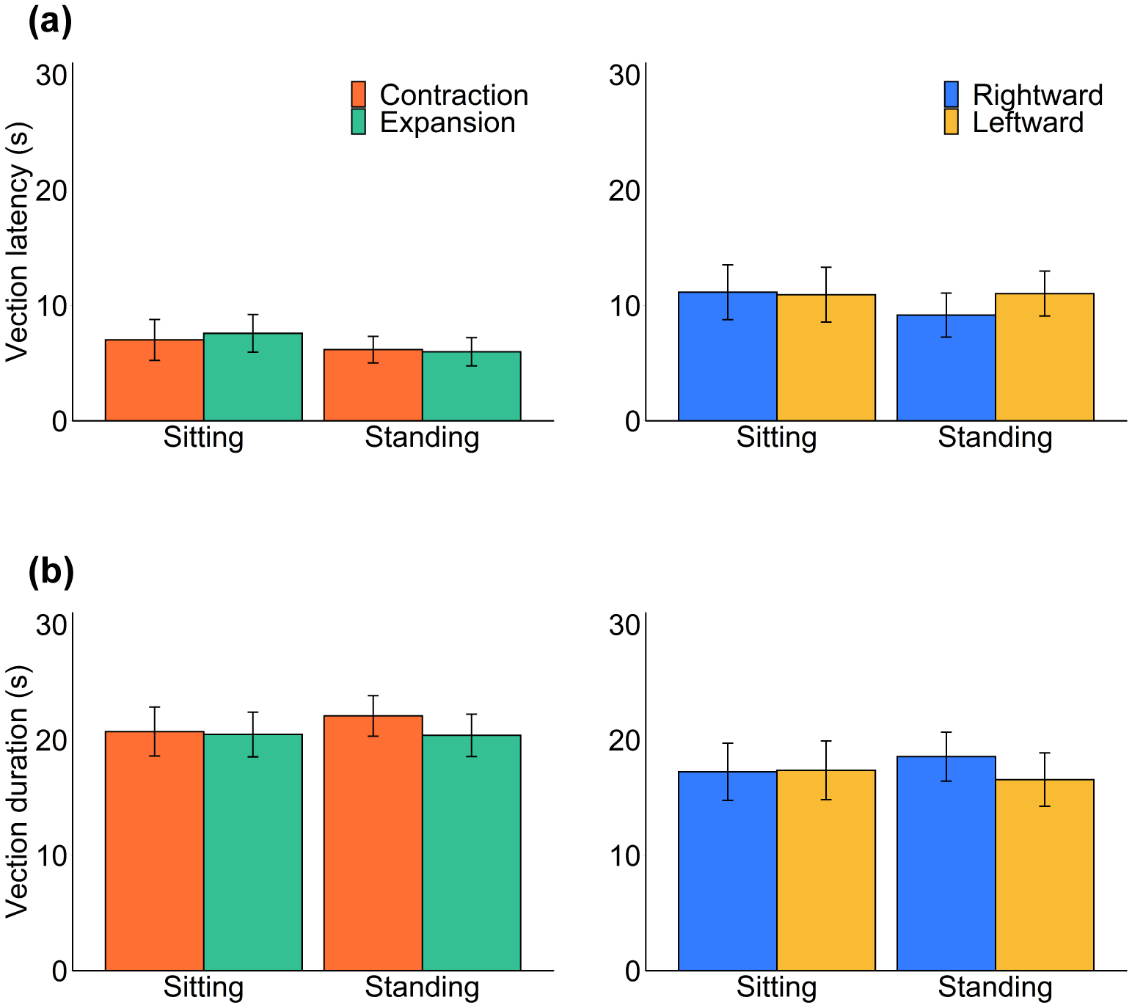


**Figure S2.** Vection results indicating mean (a) latencies and (b) durations for each posture and type of optic flow (left panel: expansion/contraction, right panel: rightward/leftward). Error bars show standard errors of the mean across participants.

**Table S1.** Pearson correlations of vection responses (rating, latency, duration) and the mean head position for each optic flow and posture.

|  | Expansion | | Contraction | | Rightward | | Leftward | |
| --- | --- | --- | --- | --- | --- | --- | --- | --- |
|  | Sitting | Standing | Sitting | Standing | Sitting | Standing | Sitting | Standing |
| Rating | 0.000 | 0.083 | -0.081 | 0.156 | -0.372 | 0.303 | -0.211 | -0.126 |
| Latency | -0.284 | -0.227 | 0.509 | -0.458 | 0.287 | -0.105 | 0.281 | -0.110 |
| Duration | -0.055 | 0.235 | -0.262 | 0.415 | -0.257 | 0.242 | -0.126 | 0.146 |

# References

Wenye, G. (2020). Detrended Fluctuation Analysis (https://www.mathworks.com/matlabcentral/fileexchange/19795-detrended-fluctuation-analysis), MATLAB Central File Exchange. Retrieved September 4, 2020.
